# Towards Standardisation of Technique for En Bloc Sacrectomy for Locally Advanced and Recurrent Rectal Cancer

**DOI:** 10.3390/jcm10214921

**Published:** 2021-10-25

**Authors:** Ailín C. Rogers, John T. Jenkins, Shahnawaz Rasheed, George Malietzis, Elaine M. Burns, Christos Kontovounisios, Paris P. Tekkis

**Affiliations:** 1 Department of Surgery, Royal Marsden Hospital, London SW3 6JJ, UK; ailinrogers@rcsi.ie (A.C.R.); Shahnawaz.Rasheed@rmh.nhs.uk (S.R.); george.malietzis@nhs.net (G.M.); paris.tekkis@rmh.nhs.uk (P.P.T.); 2 Department of Colorectal Surgery, Mater Misericordiae University Hospital, D07 R2WY Dublin, Ireland; 3 School of Medicine, University College Dublin, D04 V1W8 Dublin, Ireland; 4 Department of Surgery, St. Mark’s Hospital, Watford Road, Harrow HA1 3UJ, UK; i.jenkins@nhs.net (J.T.J.); e.burns2@nhs.net (E.M.B.); 5 Colorectal Surgical Unit, Chelsea and Westminster Hospital, Chelsea, London SW10 9NH, UK; 6 Department of Surgery and Cancer, The Royal Marsden Campus, Chelsea and Westminster Hospital and Imperial College, Paddington, London SW10 9NH, UK

**Keywords:** advanced rectal cancer, sacrectomy, en bloc, recurrent colorectal cancer

## Abstract

Treatment strategies for advanced or recurrent rectal cancer have evolved such that the ultimate surgical goal to achieve a cure is complete pathological clearance. To achieve this where the sacrum is involved, en bloc sacrectomy is the current standard of care. Sacral resection is technically challenging and has been described; however, the technique has yet to be streamlined across units. This comprehensive review aims to outline the surgical approach to en bloc sacrectomy for locally advanced or recurrent rectal cancer, with standardisation of the operative steps of the procedure and to discuss options that enhance the technique.

## 1. Introduction

Curative treatment for advanced or recurrent rectal cancer relies upon multimodality therapy with complete pathological clearance being the ultimate goal of resection. To achieve this where tumours invade or are adherent to the sacrum, en bloc sacrectomy at a radiologically predetermined level is the current standard of care. Sacral resection remains technically challenging, and the morbidity rises sequentially with higher levels of transection [1,2,3,4]. Major uncontrolled haemorrhage is a feared complication of sacrectomy, with 80% of patients shedding over a litre of blood and requiring at least one haemostatic agent [5].

Furthermore, patients universally commit to a permanent colostomy, as rectal anastomosis is almost impossible in the setting of en bloc sacrectomy with either total pelvic exenteration or abdominosacral resection. These factors, along with the low volume of patients with direct sacral invasion or adherence and an as yet unclear survival benefit from high sacrectomy have deterred many surgeons from considering the procedure as a realistic option [6].

**Figure 1:**
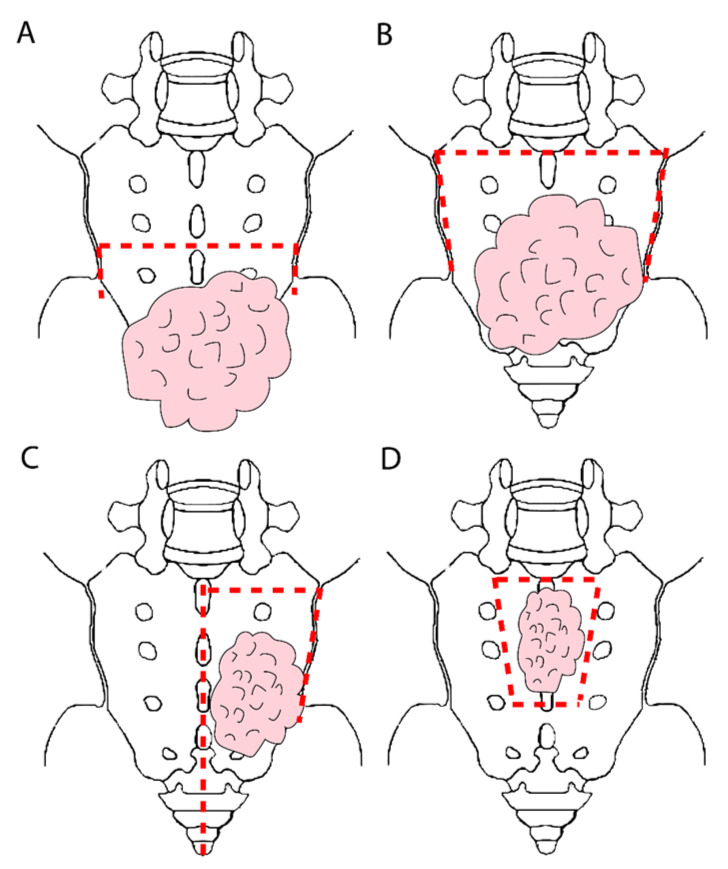
Approaches to en bloc sacral resection depending on the site of invasion of the rectal tumour. Dashed red lines denote resection margins. (**A**) Low sacrectomy involves sacral transection in the axial plane below the S2 foraminae. (**B**) High sacrectomy involves sacral transection at or above the S2 foraminae and involves disarticulation from sacroiliac joints. (**C**) Hemisacrectomy involves tumours not crossing the midline of the sacrum, thus, leaving the contralateral side intact. (**D**) High subcortical sacrectomy (HISS) or anterior table sacrectomy is reserved for tumours adherent to the median sacral plane but not involving the sacral foraminae laterally, and the anterior sacral cortex only is removed.

The types of en bloc sacral resection vary and depend on the extent of tumour involvement and surgical expertise (Figure 1), and a technical description of the operative steps involved has not yet been standardised. Sacrectomy may be categorised as high or low, based on the level of sacral transection in the axial plane, where low sacrectomy is below the foramen of the S2 nerve root, usually involving S3 or below [3]. Where tumours involve the midline of the sacrum but do not extend as laterally as the sacral foramina, a modified partial sacrectomy removing the anterior sacral cortex may be performed [7,8]. Hemisacrectomy (through the sagittal axis of the sacrum) and total sacrectomy (disarticulation at L5/S1 joint) may also be performed depending on the tumour site [9,10].

The purpose of this study is to outline the surgical approach to en bloc sacrectomy for locally advanced or recurrent rectal cancer with standardisation of the operative steps of the procedure and to discuss options that enhance the technique.

## 2. Preoperative Evaluation

Operations involving en bloc sacrectomy are superspecialist procedures, requiring multidisciplinary input, including radiology, pathology and different surgical specialties. All operations should be performed by a specialist colorectal surgeon with sacrectomy experience, with support from spinal orthopaedic/neurosurgical, gynaecological, vascular, urological and plastic surgeons on a planned individual basis. Specialist anaesthetic teams are required.

The most important decisions in the preoperative phase determine whether the oncological outcomes, morbidities and function can be balanced in a patient’s favour. Achieving clear pathological margins is mandatory. Preoperative pelvic magnetic resonance imaging (MRI) provides optimal local staging of rectal tumours and can assess the extent of sacral invasion and adherence and the relationship to surrounding structures with detailed assessment of cranio-caudal and lateral pelvic relations. The MRI images allow measurement of the distance from the sacral promontory to the required point of transection in the midline, thus, ensuring optimal tumour clearance to achieve negative margins.

This measurement should be noted for use intraoperatively. The extent of lateral dissection is also determined upon MRI. Once the decision is made as to the transection level and angle of osteotomy, patients are counselled on the surgical approach, reconstruction options and risks and benefits of the procedure, as would be standard for obtaining the informed consent for any other procedure. The related sacrifice of sciatic nerve roots and other pelvic nerves is also determined on imaging. Discussion of reconstructive options and postoperative morbidities is considered later.

## 3. Operative Steps

### 3.1. Patient Preparation and Position

Patients should undergo perioperative preparation as per standard departmental protocols and are routinely managed with perioperative antibiotics, venous thromboembolism prophylaxis and meticulous aseptic skin preparation. The patient should have a urethral catheter placed and be placed in the Lloyd–Davies position. Selective ureteric stenting is used depending on the disease extent, relationships and, hence, operative requirements. Most described approaches to en bloc sacrectomy employ an initial mobilisation through the abdominal cavity; some authors describe raising the lumbosacral junction by placing a 1 L fluid bag, rolled-up towel or manufactured gel roll behind the sacrum, elevating the perineum and allowing it to overhang the end of the operating table [11], although some choose not to employ this method.

Generally, where sacral transection is expected to be below the level of the sacroiliac joint (S3), a totally supine approach to sacrectomy is feasible and can be performed maintaining the patient in a single position with en bloc partial or total exenteration where the anus and pelvic floor and pelvic organs are excised in the Lloyd–Davies position [12]. This approach initially accesses the anterior sacrum via the abdominal cavity allowing exposure and control of the structures lying anterior and lateral to the sacrum, such as the pelvic sidewall muscles, ligaments, bony structures and the (internal) iliac vessels.

The sacral nerve roots exit their foramina travelling laterally to form the sciatic nerves fusing with the lumbar (L5) nerve roots passing through the greater sciatic notches cranial to the ischial spines and related ligaments. The anterior approach is the only approach that safely permits anterior and lateral dissection of the lumbosacral nerve roots under direct vision; the sciatic nerve exits the pelvis in a more lateral direction with the legs abducted than with the legs in the prone position [11]. Where the patient is supine, perineal reconstruction with a vertical rectus abdominis myocutaneous (VRAM) myocutaneous flap may be used, although a prone approach to reconstruction is increasingly common.

Many surgeons will choose to electively employ a prone approach for completion of low en bloc sacrectomy after an initial anterior/abdominal mobilisation phase, but where S1 or S2 are involved, and hence high en bloc sacrectomy is required, then it is an imperative to turn the patient prone after mobilisation from the anterior phase via the abdomen, to complete sacral mobilisation and en bloc tumour resection [11,13] (Table 1).

Above the S2/3 junction, the sacrum is contiguous laterally with the iliac bones that require transection in the axial and sagittal planes to complete the resection. Most patients can be safely turned to a prone position unless a significant kyphosis or chest pathology prohibits safe ventilation. Since the approach also means that pelvic access is limited, it lends an inability to gain vascular control of major intra-pelvic vessels should it be required [14], although most patients turned prone will have undergone an initial anterior mobilisation and vascular mobilisation to achieve control before turning prone.

### 3.2. Visceral Mobilisation

The pelvic organs to be resected will depend upon the disease extent and degree of organ involvement, ranging from the rectum with en bloc sacrectomy (abdominosacrectomy) to total pelvic exenteration with en bloc cystectomy, total abdominal hysterectomy, bilateral salpingectomy in women and en bloc cystoprostatectomy in men, abdominoperineal resection of the rectum and resection of any other involved abdominal or pelvic side wall structures, vessels, muscles, ligaments and bone.

Pelvic structures are usually mobilized anteriorly and laterally before the posterior or “sacral margin” is challenged with the anterior sacrum being cleared of the overlying tissues to the predetermined point of transection. The transection point should be checked by measuring down from the promontory in the midline correlating with the distance derived from preoperative MRI.

The angle of transection considers the disease extension into the sacral bone and the relationship of the planned osteotomy to the thecal sac, with attempts to avoid inadvertent cerebrospinal fluid leak. A marker (e.g., a tape strip) can be placed at the measured height on an instrument, such as a Cobb periosteal elevator, which can then be placed behind the anterior sacral periosteum to clear the anterior sacrum until the planned transection point is reached. This dissection can be extended laterally but should be first limited to within 2–3 cm on either side of the midline until the ventral foramina, nerve roots and related blood vessels are safely identified.

### 3.3. Vascular Mobilisation and Control

The presacral venous plexus and iliac vessels in the pelvic side wall are the main sources of haemorrhage encountered during en bloc sacrectomy; a number of strategies are available to minimise blood loss [15]. Bleeding from presacral veins is encountered at the point where the presacral plane is entered to expose the anterior sacral cortex and can usually be stopped with electrocautery and pressure haemostasis with a gauze pack. Haemostatic adjuncts, including oxidised cellulose or thrombin/fibrinogen sealants can be additionally used. The presacral venous plexus will ultimately be resected en bloc as part of the specimen from at least the level of the osteotomy downwards [14], and at a higher level where complete presacral fasciectomy is required.

The next step is to mobilise the vascular plane, first with ureterolysis, and then by dissecting the external iliac vessels free from their attachments. This allows external iliac artery control with a vascular sling and also reveals the medial borders of the psoas major muscles in preparation for nerve root identification. Once the L5 nerve root is identified, the internal iliac artery and vein can be identified and medialized. At this point, the branches of the internal iliac can be selectively ligated at the appropriate level. Many opt for planned iliac vessel sacrifice to avoid major bleeding; others dissect out and preserve the superior gluteal vessels to maintain gluteal blood supply.

Some groups pre-emptively embolise the iliac vessels [10]; however, most now favour intra-operative ligation in the abdominal portion of the surgery [16,17]. When adopting this approach, the arterial trunk should be ligated and divided before the veins. This may not be required for low sacrectomy but is required in almost all cases of high sacrectomy [4,18], or where cystectomy is performed [4,19]. Where cystectomy is not performed, the ligation point on the internal iliac artery should be distal to the origin of the superior vesical artery, in order to preserve the bladder arterial supply. The implications of iliac vessel ligation on the choice of myofascial flap for pelvic reconstruction are discussed below, particularly regarding preservation of the superior gluteal artery for the use of gluteal flaps.

With careful patient selection and streamlined operative techniques, loss of vascular control is exceptionally rare; however, unintended injury to the internal iliac vessels may require vascular surgeon expertise and reconstruction, or the vessels can be isolated and repaired primarily and ligated. In arterial bleeding or extremis, the aorta can be cross-clamped without heparin for 10–20 min in order to regain haemodynamic control and perfuse vital organs [20].

### 3.4. Nerve Root Identification

Prior to this step, the external iliac vessels have been mobilised medially and controlled revealing the medial borders of the psoas major muscles. The psoas’ have been mobilised along their length, starting at the promontory and moving caudally, exposing the lumbosacral plexus and sacroiliac joints behind. The sciatic nerve can be protected with a sling, although many now opt not to disrupt it in this way, with the L5 and sciatic nerve roots seen to exit their foramina and travelling laterally toward the top of the greater sciatic notch [21].

Nerve root branches below the planned transection should injected with local anaesthesia as they exit their respective foramina before ligation and sharp transection with scissors or with an energy device (Thunderbeat^®^ (TB), Olympus Medical Systems Corp., Tokyo, Japan). Any bleeding from foraminal vessels may be controlled by direct pressure and haemostatic adjuncts.

### 3.5. Sacral Division and Specimen Extraction

At S1, the sacrum can be expected to be about 2.5 cm thick, progressively thinning as it moves caudally by a half a centimetre per vertebra with a mean thickness of 2 cm and 1.5 cm through the midpoints of S2 and S3, respectively [22]. The correct angle of the transection plane required should be determined on the preoperative MRI and is usually at about 45 degrees to the operating room floor, and thus this oblique angle will confer additional sacral thickness. The relatively flat surface of the anterior cortex of S1 and S2 can be used to determine and reference the angle of transection by osteotomy. The angle of division should aim to maximally preserve bone and avoid the thecae sac without compromising the oncological resection.

Sacral division can be performed with a straight osteotome and mallet, a pneumatic drill, a Gigli saw or an ultrasonic aspirator [4,11,13,21,23,24]. Advocates of the ultrasonic bone aspirator argue that it is less traumatic with increased precision of dissection and safety in the presence of nearby vessels, ureters, dura and nerve roots [24]. With a Gigli saw, the posterior transection plane must have been cleared first in order to pass the wires behind the sacrum for sawing.

**Figure 2:**
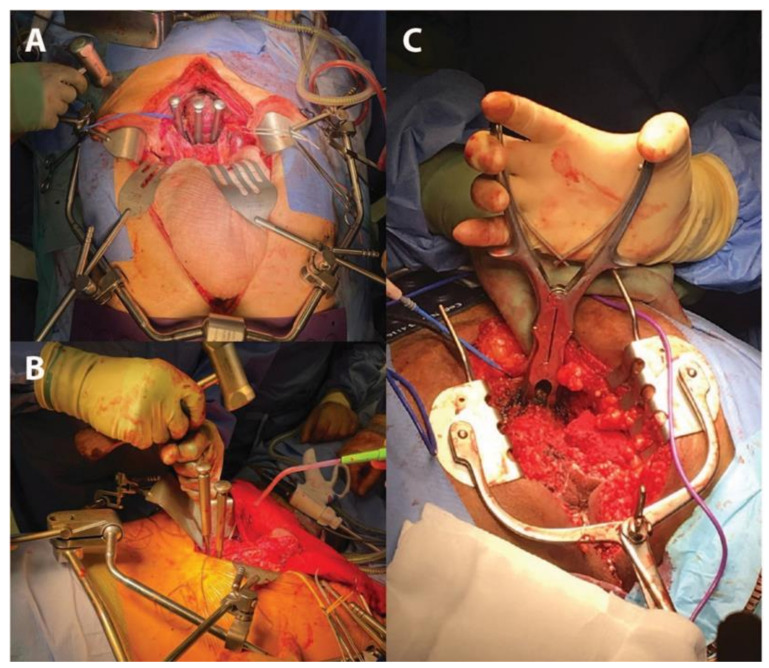
Sacral transection shown with osteotome or bone nibbler method. (**A**) and (**B**) In the supine position, osteotomes are hammered onto the desired transection level, first in the midline to breach the full thickness of the sacrum, before placing further osteotomes in series bilaterally, moving medial-to-lateral until transection has been completed. (**C**) From the prone position, the sacrum may alternatively be transected using bone nibblers.

We choose to transect anteriorly before the posterior dissection is completed, in which case an osteotome or pneumatic drill is preferable. After nerve root division, the presacral area at the level of the planned transection is now safely accessible and the anterior axial transection line should be incised with diathermy to demarcate the planned site of the osteotomy [19]. Our preferred instrument for transection is a 15 or 20 mm extended length osteotome, which is hammered onto the desired transection level at the predetermined angle from MRI assessment, commencing in the midline and breaching through the full thickness of the sacrum. A distinct drop in timbre is audible while hammering and identifies breach of the cortex posteriorly. This manoeuvre is repeated, placing further osteotomes (if available) in series lateral to the midline cut on either side, moving medial-to-lateral until transection has been completed (Figure 2A,B).

Where a patient will undergo a prone component of the dissection, some surgeons will make an entry point from the perineum into the pelvis before turning prone to ensure that the pelvis can be safely entered in the prone position to facilitate the control of bleeding and identification of structures where direct posterior entry to the pelvis on either side of the sacrum may prompt massive bleeding out of view and from an inaccessible point in the pelvis. Such a preformed entry point permits access to direct dissection and the application of pressure to bleeding surfaces.

To complete the procedure, the surgeon approaches the perineum via either the supine or prone approach. If performing this for low sacrectomy in the Lloyd–Davis position, a similar approach as for extralevator abdominoperineal excision (ELAPE) can be followed, incising elliptically and dissecting subcutaneous and ischiorectal fat to the pelvic floor, dividing the levator ani and identifying the anterior tip of the coccyx before mobilising the sacrum posteriorly. For a prone approach, an incision is made in the posterior midline, extending cranially 2–3 vertebral levels above the planned transection level and caudally to the coccyx or anus, through skin and subcutaneous tissues.

In both approaches, the gluteus muscles are reflected laterally to expose the lumbosacral fascia. For transection at S3 or above, the sacrotuberous and sacrospinous ligaments are divided at their attachments to the ischial tuberosity and spine. Palpating the ischial tuberosities and spines at this point helps to orientate the surgeon of the path of the sciatic nerve and gluteal vessels lying above and below the nerve in order to avoid injury. The piriformis muscle is split along its fibres or divided as it fuses to its tendon to access the pelvic cavity posteriorly through the endopelvic fascia.

The division of the piriformis muscle in the prone position will expose the sciatic nerve and facilitate entry to the pelvis from this point. In the prone position the erector spinae muscles are also lifted off the posterior spine and mobilised laterally to expose their junction with the transverse processes. The posterior surface of the sacrum is then exposed by removing any soft tissue overlying it, clearing as far laterally as the sacroiliac joints if necessary for transection above S3. The lateral dissection should measure 6.5 cm from the midline on each side for adequate exposure of the sacroiliac joint [22], but varies on a case-by-case basis depending on extent of resection required. Often the split posterior cortex from the previous anterior division will become evident after this component of the dissection.

When the sacrum is freed off all the attachments circumferentially and its cortex is clearly palpable, in the supine position, a flat metal instrument, such as a malleable retractor, can be placed from the perineum to lie dorsal to the sacrum and prevent overshoot and damage to soft tissues during abdominal transection of the sacrum.

When the prone approach is used, the sacral transection point from in front can be palpated with fingers [12]. Some surgeons transect only in the prone position, but this risks inadvertent visceral and vascular damage within the pelvic cavity. Where this approach is used, the transection point should identifiable from the prone position by using a marker placed abdominally from above, such as a chromium nail or Kirshner wire, which can be brought through the gluteal skin for localisation or imaged with X-rays [18]. An alternative way of transecting in the prone position is with the bone nibbler, pictured in Figure 2C, which also has the advantage of minimizing unintended injury.

**Figure 3:**
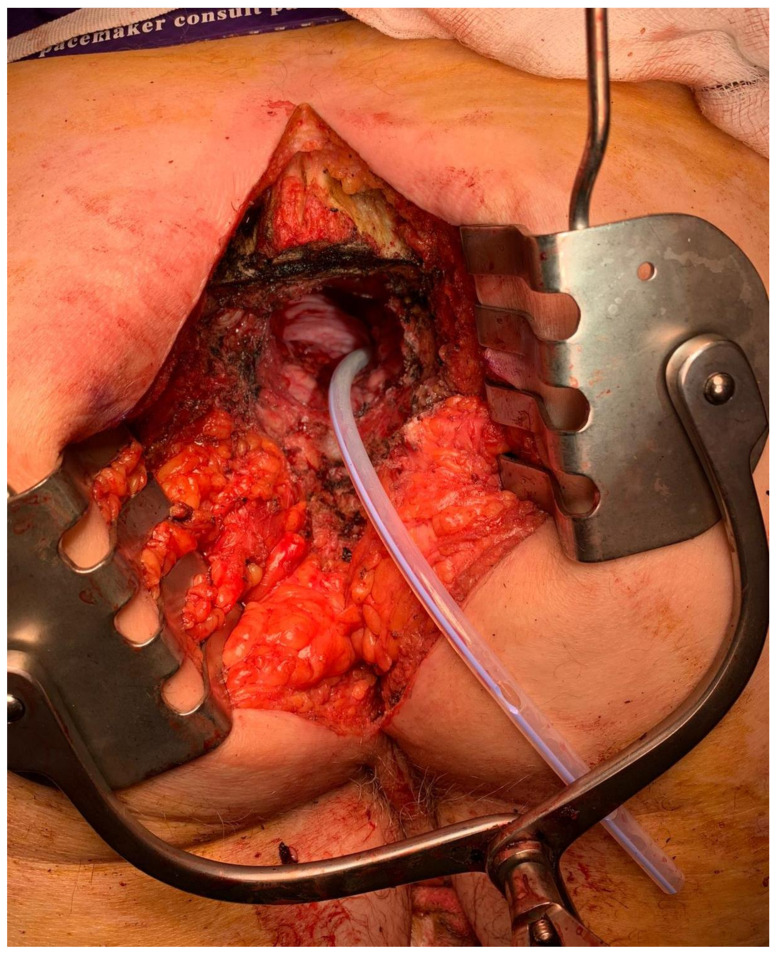
Prone view into pelvis after S3 sacral transection and specimen removal. Robinson’s drain can be seen coming from the pelvis, with St. Mark’s perineal retractor in situ.

When the sacrum is free, any remaining posterior muscular attachments are divided, the nerve roots to be sacrificed are dissected off the body of the sciatic nerve and the specimen is then delivered [11] (Figure 3). In bulky pelvic tumours where safe exposure needs to be maximised, division of the pubic symphysis to spread the pubic rami may be performed via the anterior approach. The pubis must be reapproximated and can be wired in order to heal [20].

Although bone wax can be applied as necessary over bleeding cancellous surfaces, we have avoided this in our practice. The thecal sac can be tied off just below the exiting nerve roots and transected with a scalpel [4] and should be absent below S2, as its caudal extension is to mid-body of the S2 vertebra. Others opt to not formally identify or ligate it, as the caudal aspects of the thecal sac tapers significantly in size and geometry. The preoperative MRI can be used to plan the angle of transection to avoid the thecal sac.

At this point in the surgery, with the specimen completely extracted, intraoperative radiotherapy (IORT) may be administered in a shielded operating room where a positive surgical margin is anticipated [9,25,26,27,28,29]. The wound can then be assessed for haemostasis, and the reconstruction portion of the procedure ensues.

### 3.6. Reconstruction Options

Since low sacrectomy usually involves transection below the sacroiliac joints, sacral reconstruction is not necessary. However, transection above this level may require reconstructive surgery to restore pelvic stability and can be achieved through fixation of the lumbar spine to the bony pelvis. This can be performed by primarily fixing the lumbar spine to the pelvis with titanium rods or bone allograft bone fusion [10,14], or through the use of a sacral prosthesis, such as a spacer or a 3D printed sacrum, which is then fixed to the pelvis [7,24]. These options may be best discussed with an orthopaedic specialist or surgeon with expertise in lumbosacral reconstruction. In the authors’ experience, such reconstructive options are rarely required unless transection is done at or above the mid S1 level.

The perineal defect may be reconstructed primarily, or with an omentoplasty, mesh or tissue flaps, as per the unit’s preference. The use of tissue flaps is generally favoured, in order to fill the pelvic defect, minimise the formation of postoperative pelvic haematoma and avoid radionecrosis and delayed iliac vessel haemorrhage [30]. Recurrent rectal cancer, by its nature, is commonly associated with perineal resection and subsequent reconstruction, but the addition of en bloc sacrectomy has some specific implications for the choice of tissue flap, which must be considered preoperatively.

Abdominal wall flaps, such as the vertical rectus abdominis myocutaneous flap (VRAM), are often difficult in the setting of exenteration as a result of bilateral stomas but may be favoured by those using an abdominoperineal approach. When resection is completed using the prone approach, the inferior gluteal artery perforator flap appears to be the obvious reconstructive option, using the generous volume of the gluteal muscles [31,32,33]. Their major drawback is their risk of devascularisation in the setting of internal iliac vessel sacrifice as part of high sacrectomy [7]. In such a circumstance, gluteal flaps based upon a preserved superior gluteal artery will be of utility. Where there is no significant perineal defect, the midline wound over the sacrum may be reconstructed by mobilising and approximating the gluteal muscles over the cut end of the sacrum [20].

### 3.7. Special Cases-Modified Partial Sacrectomy

A number of approaches have been described in order to resect involved sacrum en bloc but minimising the morbidity of bilateral sacral resection and nerve root sacrifice associated with sacrectomy in patients with high sacral involvement (Figure 1). Careful patient selection is paramount for these approaches and should only be undertaken where optimal oncological margin clearance is achievable.

In patients with disease limited to one side of the sacrum, not crossing the midline, a segmental hemisacrectomy has been described, excising the proximal half of one side of the sacrum and leaving the contralateral half and the distal ipsilateral half in situ. Alternatively, a hemisacrectomy in the supine position may be an option for unilaterally invading tumours [34], and this spares the joint and nerves on the contralateral side. Where disease is limited to the sacral midline and a margin is achievable without sacrificing the sacral foramina, a “high subcortical sacrectomy” (HiSS) or “anterior table sacrectomy”, is an option [7,8,35,36]. This involves resecting the involved midportion of the anterior sacral body with the rectal tumour specimen, leaving the posterior surface and lateral edges in situ [8]. This approach both optimises pelvic stability as well as avoids the neurological morbidities of dissection around the lateral edges of the sacrum.

A HiSS resection is approached from the Lloyd–Davies position after the standard visceral mobilisation is complete. Once the sacrum is accessible, the required margins for complete oncological clearance of tumour are identified. The subperiosteal plane is opened, and the periosteum cleared around the planned margins proximally and laterally. The anterior sacral body may be incised along the required margins simply using a curved osteotome to circumferentially cut through the cortex. Alternatively, holes may be burred through the anterior cortex using a bone drill around the margins of dissection, and the burr holes then joined transversely by passing the drill longitudinally into the cancellous sacral bone joining the burr holes.

A curved osteotome can then lift the marked anterior cortex of the sacrum to complete the HiSS and meet the planned distal cortical bony margin. While this approach avoids sacrifice of the S1 or S2 nerve roots, it can be used also where they are involved, with nerve root excision. An expandable spacer and bone grafts can be combined with this approach to bridge the corpectomy if required [24].

### 3.8. Minimially Invasive En Bloc Sacrectomy

Many surgeons would agree that advanced resection involving sacrectomy is difficult to complete through a minimally invasive approach. Laparoscopic en bloc sacrectomy has been described, using a Gigli saw with wires through caudal trocars to divide the sacrum [37], and a similar robotic approach may be feasible. Alternatively, the abdominal portion could be completed using a minimally invasive approach, with the sacrectomy completed using the perineal or posterior approach; however, to date, there are no reports of these [30,31,32,33].

## 4. Postoperative Complications

Complications of en bloc sacrectomy relate to the primary procedure undertaken, as well as the additional effect of the sacrectomy. In APR with flap reconstruction, complications, such as wound dehiscence, flap failure and perineal hernia, may have higher incidence when en bloc sacrectomy is additionally undertaken, with a higher transection level on the sacrum associated with higher perineal complication rates [38,39,40]. In fact, higher transections may be associated with higher all-cause morbidity, including physical function, pain, quality of life and bladder function [1,2,3,4], although other studies dispute this [13,18,41].

In patients where cystectomy and urinary diversion is not performed, sacral nerve transection or damage can lead to urinary problems postoperatively. Sacral splanchnic nerves carry parasympathetic fibres that are important in bladder function and maintenance of continence [5]. Sympathetic fibres are also important in sphincter function and bladder relaxation, especially in women [6,7]. Preservation of S2 nerve roots bilaterally can be associated with good bladder control; however, overall, most agree that preservation of one or both S3 nerve roots is optimal to avoid compromising bladder function [1].

Restoration of bowel continuity is almost unheard of in combination with sacrectomy; thus, normal bowel function and faecal continence are not goals of the surgery as standard. The sacrifice of certain sacral nerve roots will impair lower limb motor function. L5 and S1 are required for acceptable motor function and avoidance of an ipsilateral foot drop. Postoperative pain is also a significant problem for patients undergoing sacrectomy, with a subsequent impact on quality of life, irrespective of the sacrectomy level [42,43].

## 5. Conclusions

En bloc sacrectomy for locally advanced or recurrent rectal cancer remains the only definitive option in patients with sacral involvement or adherence who are considered for curative treatment. Sacrectomy type and approach is both surgeon- and patient-dependent and is a highly specialised procedure with the need for high volume throughput, MDT expertise and multispecialty input. Adequate preoperative planning and staging is paramount, as is patient counselling as to the implications of the operation. En bloc sacrectomy is a complex procedure with high levels of morbidity, and the delicate balance between the gains of curability and the drawbacks of postoperative dysfunction must be considered at every step in the patient journey.

## Figures and Tables

**Table 1:** Operative steps of en bloc sacral resection. Overview of surgical manoeuvres involved in high and low sacrectomy.

En Bloc Sacrectomy for Rectal Cancer—Operative Steps	
**Patient preparation and position**	High sacrectomy: mixed supine/prone approach required Low sacrectomy: mixed supine/prone approach or totally supine approach feasible
**Visceral mobilisation**	Start with anterolateral pelvic structures first Clear midline of anterior sacrum to predetermined transection point
**Vascular mobilisation and control**	Ureterolysis and external iliac mobilisation External iliac artery control Dissect free the medial psoas borders Identify L5 and control internal iliac artery Selective internal iliac branch ligation
**Nerve root identification**	Identify sciatic nerve roots exiting their foramina below L5 Transect branches below planned sacral division
**Sacral division and specimen extraction**	Extend clearance of anterior sacral cortex laterally to planned transection line Diathermy mark transection level Osteotome x 3 through premarked point, starting midline and then bilaterally Alternatively use pneumatic drill or Gigli saw Divide posterior sacral soft tissue attachments and remove via perineal or prone approach Haemostasis
**Reconstruction**	Visceral restoration of continuity/ostomy +/− omental flap/mesh for pelvic defect +/− bony reconstruction Myofasciocutaneous flaps, most commonly now raised on the superior gluteal arteries

## Data Availability

Not applicable.
